# Partial epilepsy: A pictorial review of 3 TESLA magnetic resonance imaging features

**DOI:** 10.6061/clinics/2015(09)10

**Published:** 2015-09

**Authors:** Lucas Giansante Abud, Lionel Thivard, Thiago Giansante Abud, Guilherme Seizem Nakiri, Antonio Carlos dos Santos, Didier Dormont

**Affiliations:** I Hospital das Clínicas da Faculdade de Medicina de Ribeirão Preto da Universidade de São Paulo, Neuroradiology, Ribeirão Preto/, SP,, Brazil; II Hôpital de laPitié-Salpêtri`re, Neurology/Neuroradiology, Paris, France; III Universidade Federal de São Paulo, Neuroradiology, São Paulo/, SP,, Brazil

**Keywords:** Magnetic Resonance Imaging, 3 T, Partial Epilepsy

## Abstract

Epilepsy is a disease with serious consequences for patients and society. In many cases seizures are sufficiently disabling to justify surgical evaluation. In this context, Magnetic Resonance Imaging (MRI) is one of the most valuable tools for the preoperative localization of epileptogenic foci. Because these lesions show a large variety of presentations (including subtle imaging characteristics), their analysis requires careful and systematic interpretation of MRI data. Several studies have shown that 3 Tesla (T) MRI provides a better image quality than 1.5 T MRI regarding the detection and characterization of structural lesions, indicating that high-field-strength imaging should be considered for patients with intractable epilepsy who might benefit from surgery. Likewise, advanced MRI postprocessing and quantitative analysis techniques such as thickness and volume measurements of cortical gray matter have emerged and in the near future, these techniques will routinely enable more precise evaluations of such patients. Finally, the familiarity with radiologic findings of the potential epileptogenic substrates in association with combined use of higher field strengths (3 T, 7 T, and greater) and new quantitative analytical post-processing techniques will lead to improvements regarding the clinical imaging of these patients. We present a pictorial review of the major pathologies related to partial epilepsy, highlighting the key findings of 3 T MRI.

## INTRODUCTION

Epilepsy is a public health problem that afflicts more than 50 million people worldwide 1. This common chronic neurological disorder is characterized by recurrent, unprovoked seizures that occur because of acute systemic or neurological insult 2. Epilepsy syndromes are categorized as either idiopathic generalized epilepsy involving a diffuse origin or partial epilepsy including symptomatic and cryptogenic types. When possible, magnetic resonance imagining (MRI) is performed to diagnose focal epilepsy. Epilepsy typically requires long-term pharmacotherapy; however, medical management remains unsatisfactory for approximately one third of patients with partial epilepsy. Neurosurgical intervention might be an effective therapy for selected cases 3,4. MRI has emerged as the most valuable tool for the preoperative localization of the epileptogenic lesion because of its excellent soft-tissue contrast. Moreover, its sensitivity at 1.5 T for patients with medically refractory partial epilepsy is between 82% and 86% 5,6. The detection of a lesion that might correspond with the epileptogenic zone enables an etiological diagnosis of symptomatic versus cryptogenic epilepsy. In certain patients, newly diagnosed epileptic seizures lead to the discovery of an evolving lesion (e.g., a neoplasm, infectious disease or immune encephalitis), which might require a specific surgical or medical treatment independent of epilepsy management.

Furthermore, in the case of patients with refractory epilepsy and a clearly delineated lesion (e.g., those with mesial temporal lobe epilepsy or hippocampal sclerosis), a preoperative evaluation via scalp video-EEG is often sufficient to identify the epileptogenic zone, thereby resulting in a positive postoperative prognosis. On the other hand, patients who suffer from refractory partial epilepsies without lesions detected using structural MRI (the so-called “cryptogenic” epilepsies) are not ideal candidates for surgery because their pre-surgical evaluation is often complicated 7 and requires expensive and time-consuming procedures involving intracranial EEG recording; furthermore, their post-surgical outcomes are frequently unsatisfactory.

Recent studies have shown that 3 T MRI has great potential for improving the detection and characterization of structural lesions compared with 1.5 T because of its greater signal-to-noise ratio. This ratio translates into higher image resolution that facilitates the detection of subtle epileptogenic lesions 8-14. Faced with this evidence, we assume that 3 T MRI is preferable when patients with refractory epilepsy have a negative or equivocal 1.5 T scan (especially when a focal cortical dysplasia is suspected). Furthermore, we recommend that 3 T systems be used routinely in clinical investigations of epileptogenic substrates.

In addition, advanced MRI techniques with quantitative evaluations such as diffusion tensor imaging, proton spectroscopy, magnetization transfer imaging, T2 relaxometry, double inversion recovery imaging and thickness and volume measurements of cortical gray matter have been applied to this group of patients to improve the detection of epileptogenic substrates. These new techniques and image processing methods are being applied to improve the detection of epileptogenic substrates, especially for cases when conventional MRI does not clearly depict a lesion 15-19.

All patients underwent a 3 T MRI examination with an HDX 3 T MRI (General Electric Healthcare, Milwaukee, Wisconsin, USA) at Pitié-Salpêtrière Hospital, Paris, France or with an Achieva Duo 3 T (Philips Medical Systems, Best, Netherlands) at the Faculty of Medicine of Ribeirão Preto, University of São Paulo, Ribeirão Preto, SP, Brazil according to the most appropriate protocol. A systematic approach was applied to MRI interpretation. Routine MRI in patients with epilepsy at these institutions includes an axial turbo spin-echo T2-weighted sequence; a coronal turbo spin-echo T2-weighted sequence perpendicular to the long axis of the hippocampus; a coronal fluid-attenuated inversion recovery (FLAIR) image perpendicular to the long axis of the hippocampus; an axial T2*-weighted gradient-echo sequence or susceptibility-weighted imaging (SWI); and a T1-weighted volume fast spoiled gradient recalled (FSPGR-IR) sequence. Contrast administration was performed depending on the clinical situation, particularly for neoplasm evaluation in addition to more advanced techniques in select cases.

This article retrospectively reviews the 3 T MRI features of various pathologic entities in a sample of patients with partial epilepsy, with an emphasis on the importance of this imaging method regarding the detection of epileptogenic lesions.

## MESIAL TEMPORAL SCLEROSIS

Hippocampal sclerosis, which is characterized by gliosis and neuronal loss, is the most common cause of medically refractory epilepsy. MRI is a useful tool for depicting hippocampal abnormalities 20-23. Patients with this condition often have a history of childhood febrile seizures followed by a seizure-free interval of several years before the onset of often medically intractable recurrent seizures.

**Figure 1:**
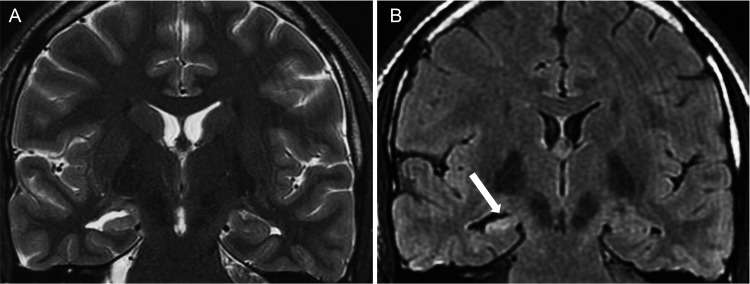
Hippocampal sclerosis in a 31-year-old man with refractory right mesial temporal sclerosis. A) coronal T2-weighted image and B) coronal FLAIR images, both perpendicular to the long axis of the hippocampus. These images show a hyperintense signal, the loss of digitations and atrophy in the right hippocampal head (white arrow).

Coronal sequences perpendicular to the long axis of the hippocampus are the best approach to visualize mesial temporal sclerosis. These sequences demonstrate a hyperintense signal on T2-weighted images and hippocampal atrophy (Figure 1). Another diagnostic criterion used to establish the MRI diagnosis of hippocampal sclerosis is the complete loss of digitations in the hippocampal head, with a sensitivity of 92% and a specificity of 100% 21. Associated findings include increased T2 signal in the anterior temporal lobe white matter, atrophy of the fornix and mammillary bodies and atrophy of the parahippocampal gyrus.

**Figure 2:**
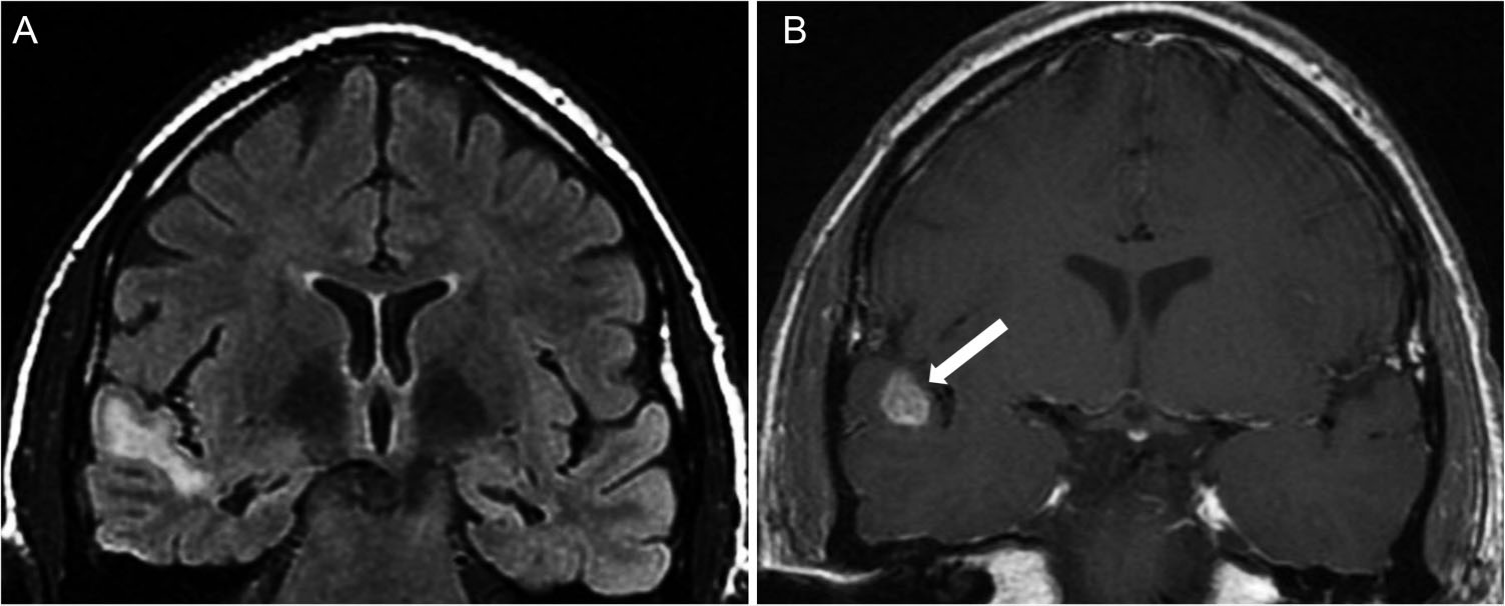
Bilateral hippocampal sclerosis and “dual pathology” in a 34-year-old woman. **A)** Coronal FLAIR image showing bilateral hippocampal atrophy and hyperintensity as well as a hyperintense lesion in the right superior temporal gyrus. **B)** Coronal T1-weighted image after a contrast administration revealed a nodular mass enhancement situated at the gray-matter-white-matter junction (white arrows); this mass might correspond to a developmental neoplasm. This lesion appeared stable on consecutive MRI scans.

Bilateral hippocampal sclerosis occurs in approximately 10 to 20% of cases and can be difficult to identify using MRI. In these cases, quantitative methods and the identification of the complete loss of digitations in the hippocampal head are useful for diagnosis (Figure 2A).

The so-called “dual pathology” occurs when mesial temporal sclerosis is associated with another epileptogenic abnormality, and it is observed in 8-22% of patients with surgical epilepsy. Thus, an exhaustive search for additional epileptogenic lesions must be performed, even after the detection of a possible epileptogenic substrate using MRI 24 (Figure 2).

Temporal lobe epilepsy is potentially curable with surgery; however, this cure depends on the accurate identification of hippocampal sclerosis on MRI scans. Recent studies have found that improved imaging of the internal structures of the hippocampus using 3 T MRI have acceptable histopathological correlations, which suggests that this scanning technique can be used to detect mesial temporal sclerosis and predict postoperative seizure outcomes in this group of patients 12,13. Moreover, 3 T MRI scans assessed using the quantification of hippocampal volume and signal can improve the detection of epileptogenic substrates, even among experienced radiologists 14.

## CORTICAL DEVELOPMENT MALFORMATIONS

Cortical development malformations account for 10-50% of all pediatric epilepsy cases and 4-25% of all adult cases. These malformations can be classified into four categories: malformations caused by abnormal neuronal and glial proliferation or apoptosis; those caused by abnormal neuronal migration; those caused by abnormal neuronal organization; and malformations of cortical development not otherwise classified 25-27. However, the most common cortical development malformations encountered in these patients is focal cortical dysplasia.

**Figure 3:**
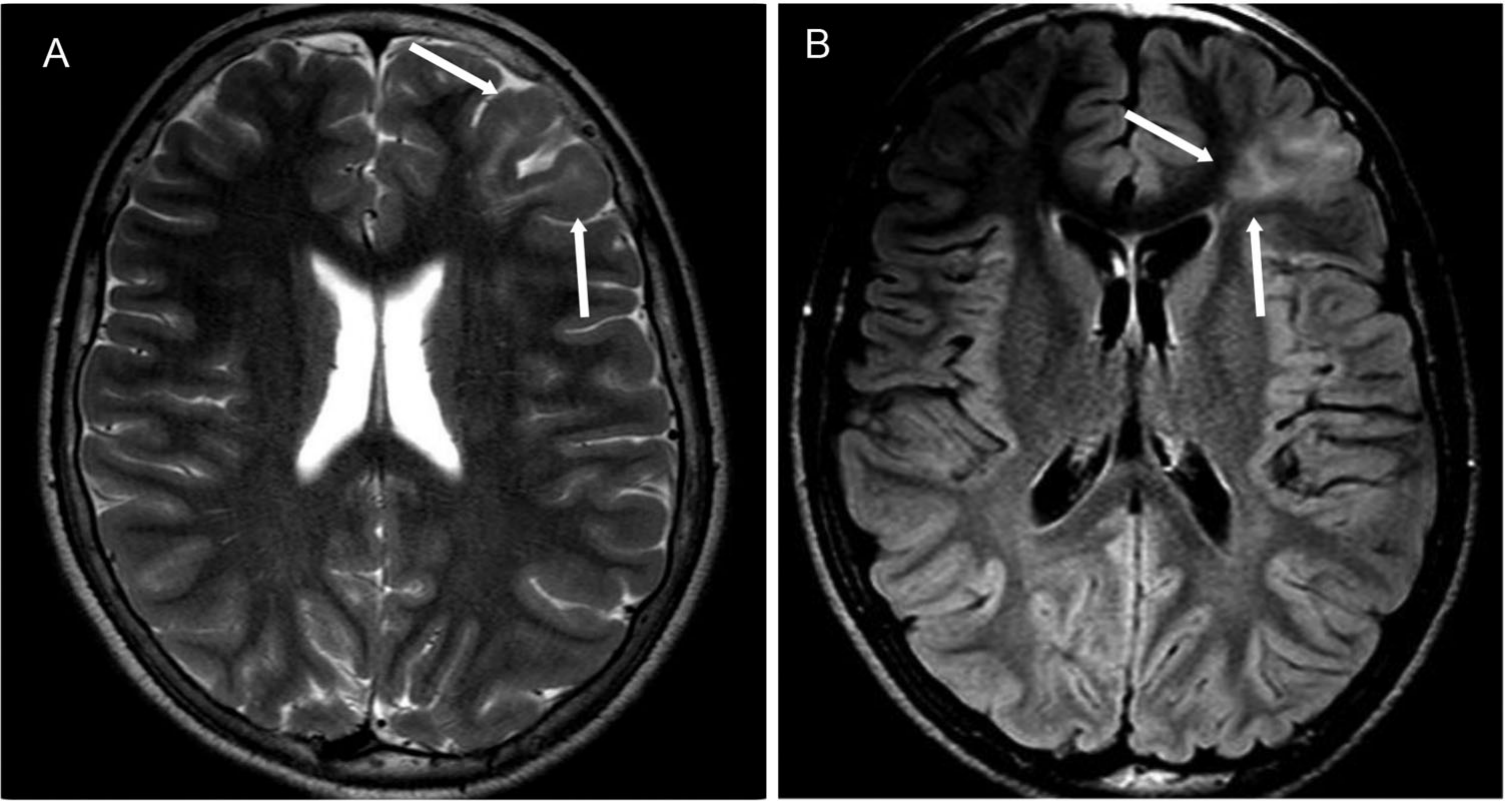
A typical MRI of focal transmantle cortical dysplasia in a 34-year-old woman. **A)** A coronal T2-weighted image demonstrating focal hyperintensity and the thickening of the right frontal cortex with a loss of demarcation between the gray and white matter (white arrow). **B)** A coronal T2 FLAIR showing a band of abnormal signal intensity extending from the cortex to the lateral ventricle (white arrows).

The advent of MRI has revolutionized our understanding and recognition of the malformations caused during cortical development. Because many of these defects are subtle, the use of high-resolution 3 T imaging (which enables excellent visualization of the corticomedullary junction) might be necessary 8-11. The term “focal cortical dysplasia” denotes a spectrum of laminar structure cortical abnormalities variably associated with giant neurons, dysmorphic neurons and balloon cells 28-30. MRI findings best evaluated at 3 T include local cortical thickening, morphological surface alterations, the blurring of the grey-matter to white-matter junction, the T2 and FLAIR hyperintensities of gray and white matter and the bands of abnormal signal intensity from the cortex to the lateral ventricle. Increased signal in the underlying white matter that sometimes appears as a wedge-shaped tail extending radially to the ventricle is also known as “transmantle dysplasia” (Figure 3). Other cortical development malformations include the agyria-pachygyria spectrum, gray matter heterotopia, polymicrogyria and schizencephaly 31-33.

**Figure 4:**
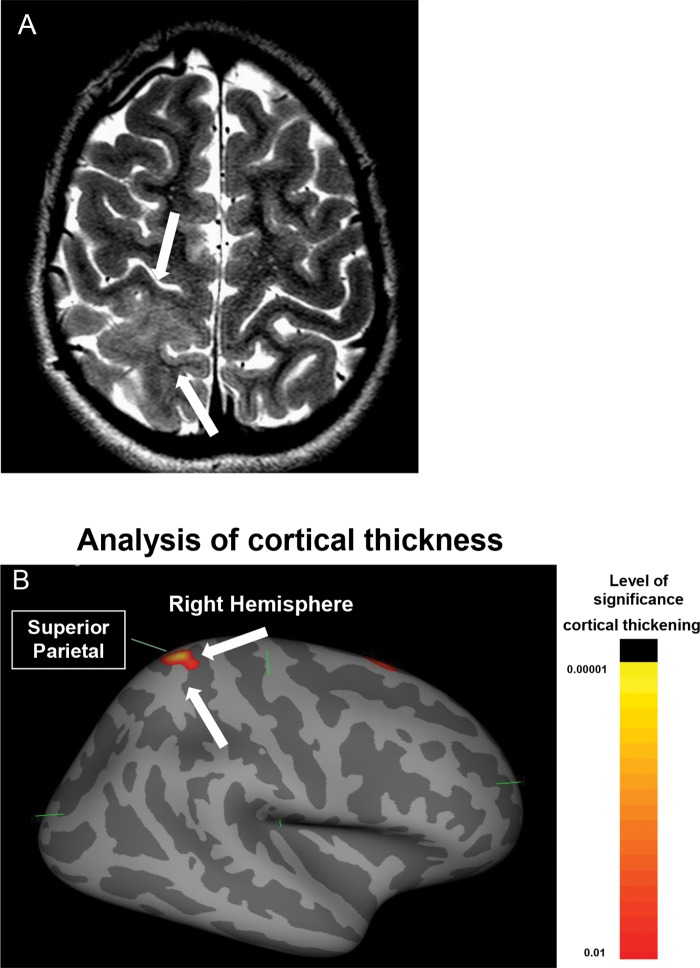
Focal cortical dysplasia type IIB of Palmini in a 10-year-old girl. **A)** An axial T2-weighted image demonstrating a focal thickening of the right parietal cortex (white arrows). **B)** An automated analysis of cortical thickness generated by Freesurfer using a volumetric T1-weighted image. These measurements clearly show a significant focal area of cortical thickening in the right superior parietal region that corresponds to the lesion observed in the T2-weighted sequence.

A recent study analyzed Type 2 focal cortical dysplasia imaging using five criteria: cortical thickening, blurring, cortical signal changes, subcortical signal changes, and “transmantle” signature. This study showed that 3 T MRI improves the detection and characterization of these lesions compared with 1.5 T MRI, which corroborates other studies in the literature 8-11. In addition, the use of advanced techniques such as cortical thickness measurements and diffusion tensor imaging can assist in the detection of areas of focal cortical dysplasia in some cases 15-19. We can automatically calculate cortical thickness from high-resolution 3D T1-weighted gradient-echo images using FreeSurfer software (Athinoula A. Martinos Center for Biomedical Imaging at Massachusetts General Hospital, Massachusetts, USA; Figure 4).

## TUBEROUS SCLEROSIS

**Figure 5:**
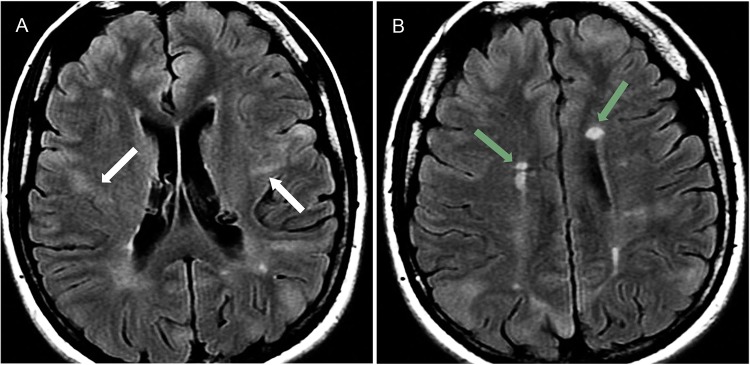
Tuberous sclerosis in a 21-year-old female with drug-resistant partial epilepsy. **A)** Axial FLAIR images show hyperintense cortical tubers with a band of abnormal signal intensity from the cortex to the lateral ventricle (white arrows) and **B)** multiple subependymal hamartome (green arrows) with a hyperintense signal.

Tuberous sclerosis is an autosomal dominant disorder that results in multiorgan hamartome. Classically, this disease is presented as a triad of clinical features (i.e., the Vogt triad): mental retardation, epilepsy, and adenoma sebaceum. The central nervous system involvement of tuberous sclerosis is characterized by cortical tubers, subependymal nodules and subependymal giant cell astrocytomas. Cortical tubers are developmental abnormalities of the cerebral cortex that are characterized by loss of the normal six-layer structure of the cortex and the presence of dysmorphic neurons and large astrocytes. Cortical tubers are histologically and radiologically identical to focal cortical dysplasia with balloon cells, also known as “forme-frusta tuberous sclerosis” 34 (Figure 5).

## NEOPLASMS

**Figure 6:**
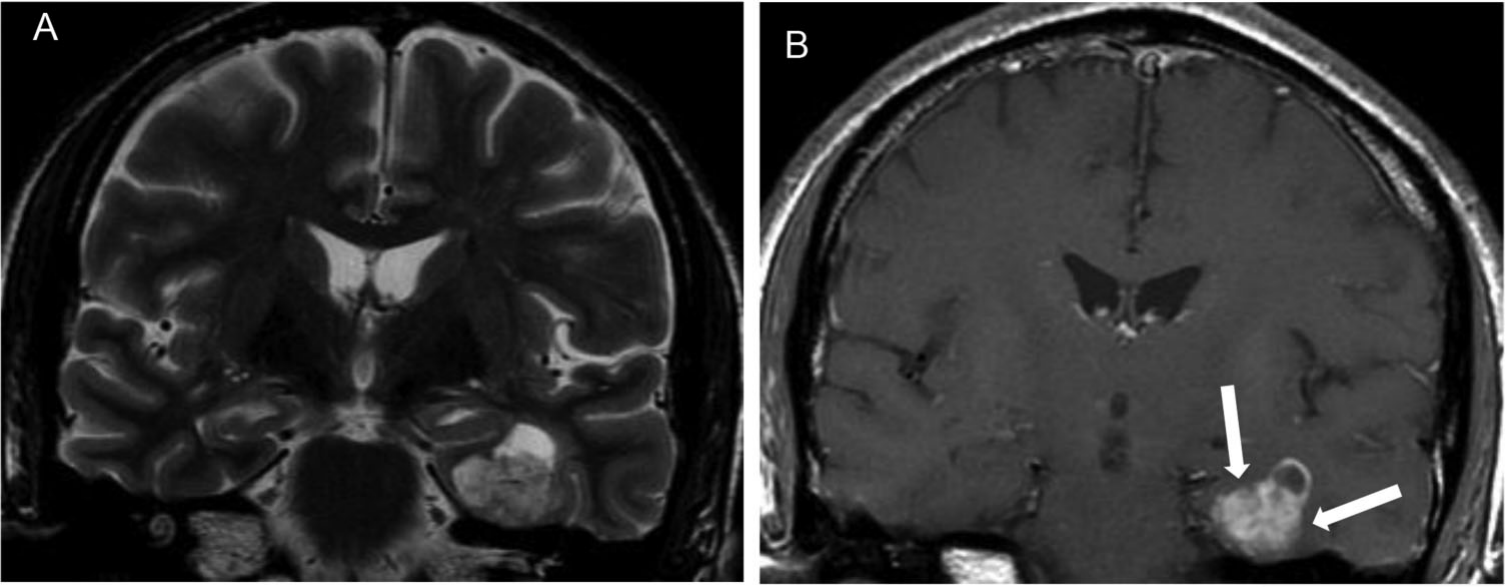
A MRI of a ganglioglioma in a 45-year-old man with refractory left partial mesial temporal lobe epilepsy. **A)** A coronal T2-weighted image and **B)** a contrast-enhanced coronal T1-weighted image showing a solid-cystic lesion in the mesial left temporal lobe with intense enhancement of the soft tissue component (white arrows).

Neoplasms associated with partial epilepsy constitute a distinct group of tumors that are often revealed by seizures in young patients. These neoplasms are located in the cortex (typically in the temporal lobe) and can be difficult to differentiate from cortical dysplasias 35,36. Gangliocytoma, dysembryoplastic neuroepithelial tumors and ganglioglioma are currently considered as cortical development malformations secondary to abnormal stem cell proliferation 17. These developmental neoplasms appear as focal lesions on MRI, often with a cystic component, and are generally cortical (Figure 6). Glial neoplasms can also be responsible for intractable epilepsy.

## VASCULAR MALFORMATIONS

**Figure 7:**
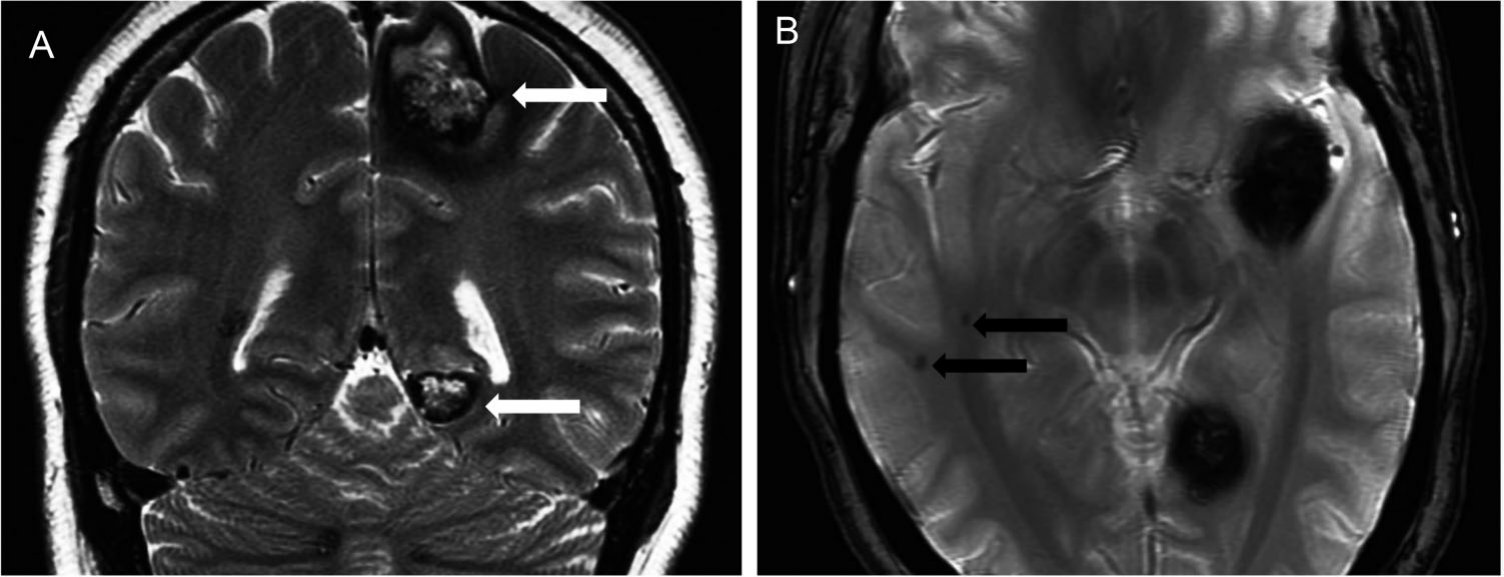
Familial cerebral cavernous malformations caused by a KRIT1 gene mutation in a 36-year-old woman with multiple epileptic foci (video-EEG) and chronic partial epilepsy. **A)** Coronal T2-weighted images show the typical appearance of a type 2 cavernoma (white arrows) characterized by mixed signal intensity with a hemosiderin ring. **B)** An axial T2*-weighted gradient-echo sequence revealing two type 4 cavernomas (black arrows) in the right temporal lobe, only observed using this sequence.

Cavernous malformations are often associated with intractable focal epilepsy, and surgical resection is appropriate in the case of a unique lesion 37. Cavernomas usually occur sporadically; however, they can be hereditary and occur after radiation therapy. Cavernomas are best detected using MRI. Four distinct appearances have been described. Type 1 lesions correspond to subacute hemorrhage and appear hyperintense using T1 sequences but hyper- or hypointense using T2 sequences. Type 2 lesions have a well-delineated complex reticulated core of mixed signal intensities representing multiple hemorrhages of varying age, and a complete hypointense rim of hemosiderin has been noted on T2 images. Type 3 lesions are iso- hypointense according to T1 sequences but hypointense on T2 sequences. Lastly, type 4 lesions are only observed using gradient echo or SWI sequences as punctuate hypointense areas 38 (Figure 7).

## MISCELLANEOUS PATHOLOGIES WITH GLIOSIS

**Figure 8:**
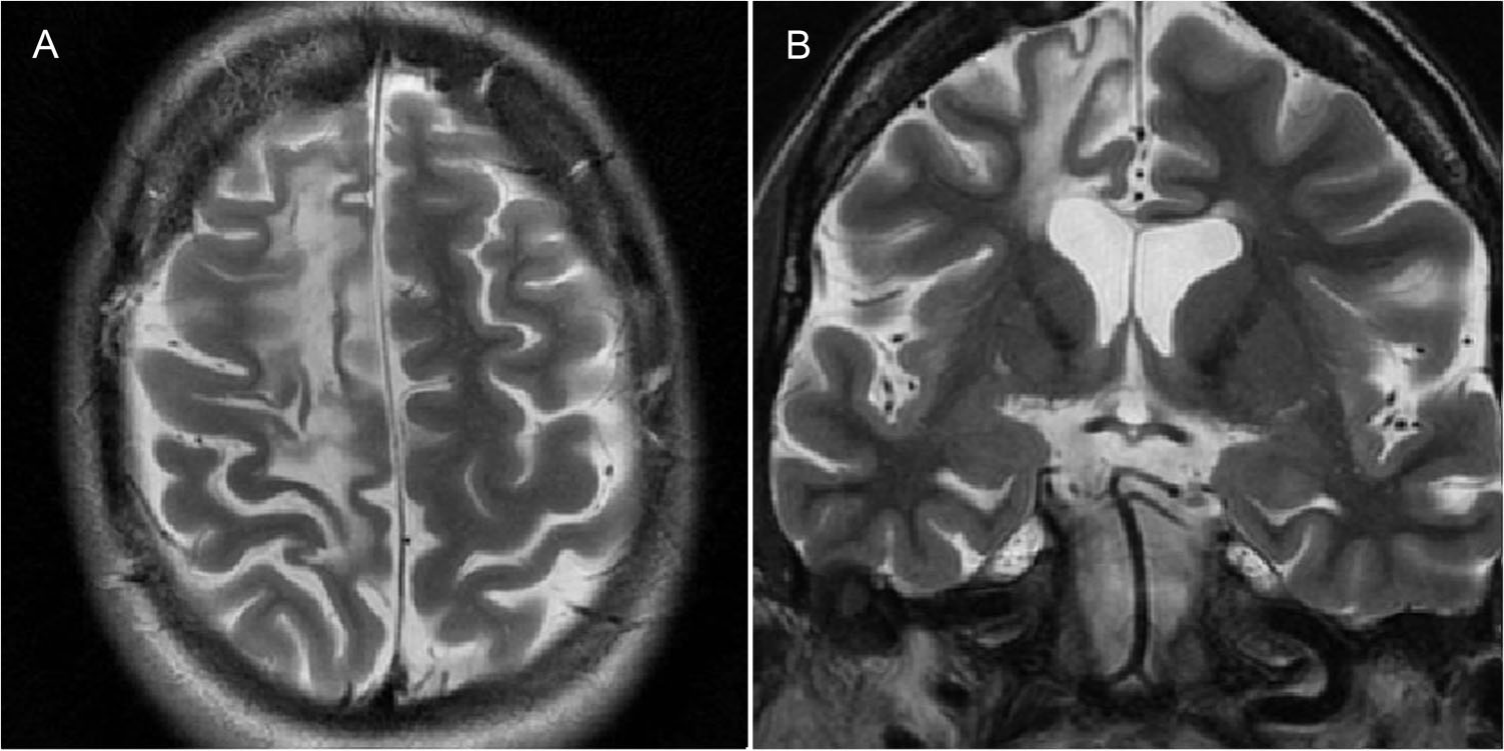
Rasmussen encephalitis confirmed via biopsy in a 37-year-old woman with refractory partial motor epilepsy. **A)** An axial T2-weighted image and **B)** a coronal T2-weighted image showing atrophy and gliosis of the right frontal lobe.

**Figure 9:**
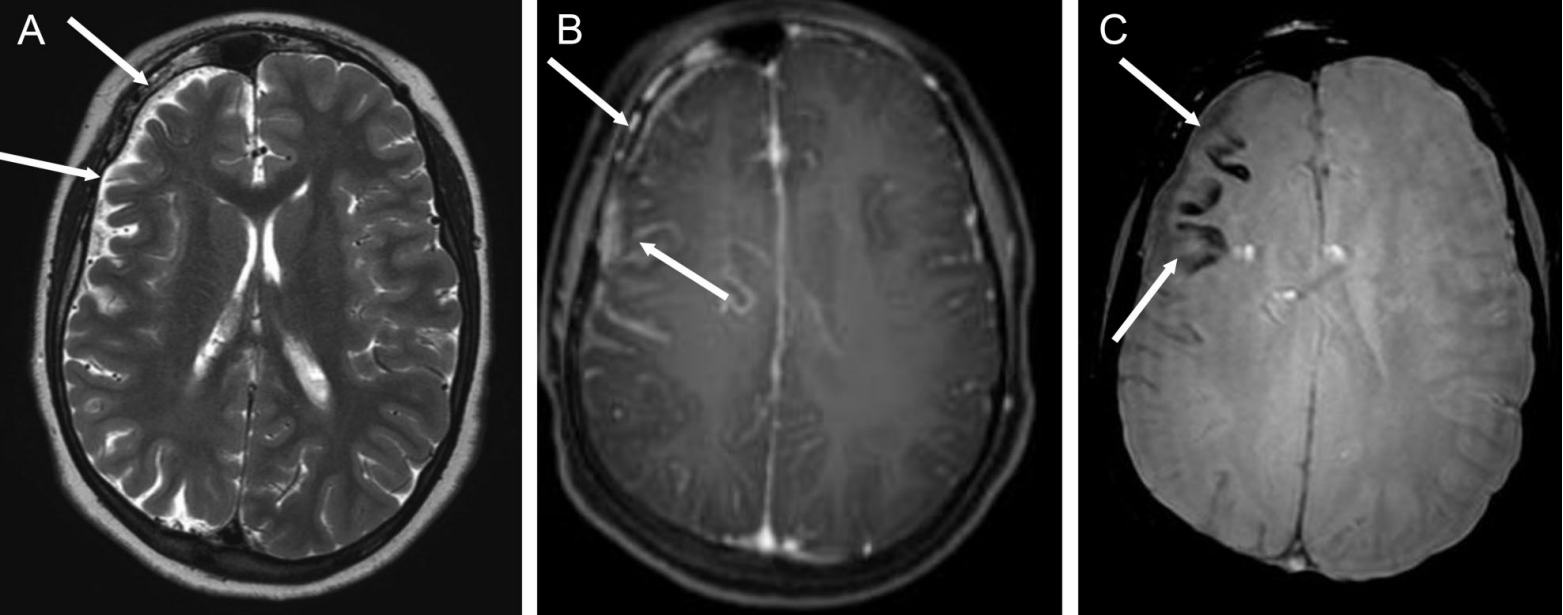
Sturge-Weber syndrome in a 14-year-old boy with multiple epileptic foci in the right cerebral hemisphere (video-EEG) and chronic partial epilepsy. **A)** An axial T2-weighted image showing right cerebral hemiatrophy (white arrows). **B)** An axial T1-weighted sequence after gadolinium injection, demonstrating leptomeningeal enhancement of the fronto-parietal region (white arrows). **C)** Axial SWI revealing cortical calcification in the frontal lobe, appearing as signal voids (white arrows).

**Figure 10:**
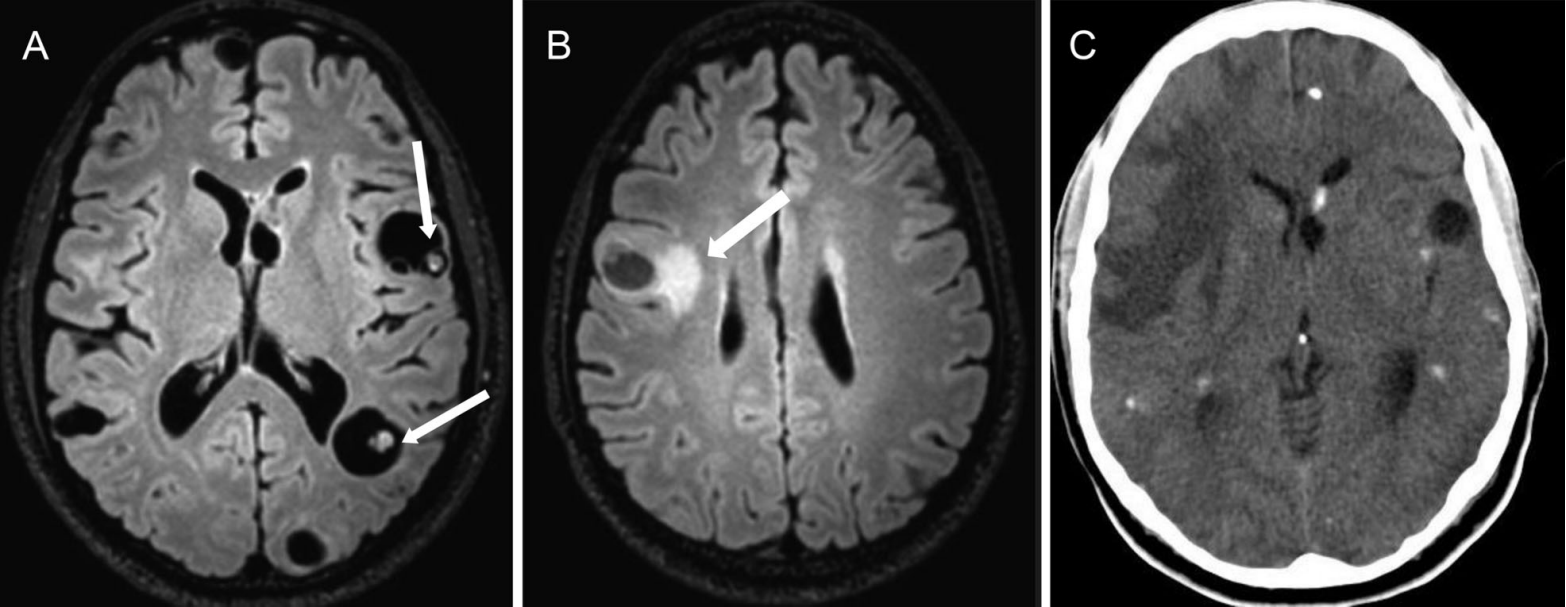
Disseminated parenchymal neurocysticercosis in a 23-year-old man with intractable epilepsy and multiple epileptic foci (video-EEG). **A)** An axial T2 FLAIR sequence showing multiple vesicular cysts, some with scolex inside (white arrows). **B)** An axial T2 FLAIR image revealing perilesional edema in the right frontal lobe (white arrow). **C)** An axial TC shows multiple calcified lesions. These findings characterize the different phases of neurocysticercosis, which are pathognomonic of this disease.

1. Rasmussen encephalitis is a chronic childhood disease, possibly of autoimmune origin, characterized by progressive inflammation of the brain, seizures and psychomotor deterioration 39. Initial imaging can be normal; over time, however, T2 prolongation appears in the cerebral cortex and subcortical white matter on MRI. Atrophy can develop during the chronic phase 40 (Figure 8).
2. Sturge-Weber syndrome consists of a facial port wine nevus in the trigeminal nerve distribution, leptomeningeal angiomatosis, epilepsy and mental retardation. Characteristic tram-track gyriform calcification is observed on CT scans, and this condition appears as a linear low signal on MRI associated with atrophy of the involved hemisphere 41 (Figure 9).
3. Posttraumatic seizures are the result of brain damage caused by physical trauma and might be a risk factor for posttraumatic epilepsy. This physiopathology includes cortical gliosis and the deposition of hemosiderin (a potent epileptogenic agent) 42. Subsequent studies have shown that cerebrovascular disease is the most common cause of seizures in the elderly, and the pathologic mechanism of chronic epilepsy after stroke is similar to that of posttraumatic epileptogenesis 43.
4. Central nervous system infections can cause chronic epilepsy due to gliotic scarring. Neurocysticercosis is a potential cause of chronic epilepsy in developing countries, and this condition is usually associated with calcified lesions. However, this cause of intractable epilepsy is uncommon even in endemic regions and might represent a comorbid pathology in most patients 44 (Figure 10).

## CONCLUSION

MRI is one of the most valuable tools for preoperative localization of epileptogenic lesions. Due to their large variety of presentation, including subtle imaging characteristics, their analysis requires careful and systematic MRI interpretation. The precise characterization of these images is crucial for the correct diagnosis and management of these patients. We presente a pictorial review of the main pathologies related to partial epilepsy, highlighting their key findings at 3 T MRI imaging.

Furthermore, several studies showed that MRI at 3 T performed better than 1.5-T in image quality, detection and characterization of structural lesions, indicating that high-field-strength imaging should be considered for patients with intractable epilepsy and normal or equivocal findings on 1.5 T MRI, who could benefit from surgery. In parallel, advanced MRI techniques with post-processing and quantitative analysis, such as thickness and volume measurements of cortical gray matter, have emerged as a near future possibility to be used routinely and allow more precise evaluation in this group of patients. Finally, we can assume that the combined use of higher field strengths (3 T, 7 T, and greater) and new sequences, associated or not with quantitative analytical post-processing, means the new directions in clinical imaging of epileptogenic substrates.
